# Central adiposity and α-klotho: inflammatory mechanisms underlying aging biomarkers related to body roundness index

**DOI:** 10.1186/s12944-025-02541-6

**Published:** 2025-04-10

**Authors:** Rui Du, Xiaoyan Tang, Lei Guan, Yuchen Lai, Huijuan Xiang, Wei Huang

**Affiliations:** 1 Department of Ultrasound, General Hospital of Central Theater Command, No. 627, Wuluo Road, Hubei, 430070 Wuhan China; 2 Department of Cardiology, General Hospital of Central Theater Command, No. 627, Wuluo Road, Hubei, 430070 Wuhan China; 3 School of Medicine, Wuhan University of Science and Technology, No. 2, Huangjiahu, West Road, Hubei, 430065 Wuhan China

**Keywords:** α−Klotho, Aging, Obesity, Body roundness index, NHANES

## Abstract

**Background:**

Obesity is a global health issue which has been widely accepted as an aging related pathogenesis. α-Klotho is a protein involved in aging process, mineral metabolism, insulin sensitivity, and the pathogenesis of various age-related diseases. Adiposity correlates with lower soluble α-Klotho, but the role of fat distribution and inflammation remains unclear. The body roundness index (BRI) refines central adiposity assessment beyond BMI. Herein, We aimed to investigate the relationship of BRI, inflammation and serum level of soluble α-Klotho.

**Methods:**

We conducted a cross-sectional analysis of 9,958 U.S. adults (40–79 years) from the 2007–2016 NHANES. We examined association between BRI and serum α-Klotho (SαKl) levels, controlling for demographic, socioeconomic, lifestyle, and clinical factors. We also assessed whether inflammatory markers mediated the BRI–SαKl relationship.

**Results:**

BRI was inversely associated with SαKl levels (*P* < 0.05). A significant sex interaction was found (*P* < 0.001), while BRI was positively correlated with multiple proinflammatory markers, which were all inversely related to SαKl levels. Mediation analyses showed inflammatory markers accounted for 20.5% (WBC), 18.0% (neutrophils), and 12.3% (platelets) of the BRI–SαKl association.

**Conclusion:**

More severe central adiposity measured by BRI was related to lower SαKl, which may partly be attributed to inflammation. These findings underscore the importance of fat distribution and inflammation in obesity-related aging and may guide interventions to preserve SαKl levels. Longitudinal studies are needed to confirm causality and inform future strategies.

**Supplementary Information:**

The online version contains supplementary material available at 10.1186/s12944-025-02541-6.

## Introduction

Obesity is a global health concern linked to a myriad of chronic conditions, including cardiovascular disease (CVD), diabetes, chronic low-grade inflammation, and accelerated aging [1]. Traditional measures of adiposity, such as Body Mass Index (BMI), often fail to capture body fat distribution and overall metabolic risk [2]. The Body Roundness Index (BRI), developed to incorporate waist circumference into an anthropometric assessment of body shape, provides a more refined estimation of central adiposity and better reflects metabolic risk than BMI alone [3–5]. Moreover, growing evidence suggests a strong association between BRI and inflammatory markers, including C-reactive protein and interleukin-6, implying that greater body roundness may drive systemic inflammation [6].

α-Klotho (αKl), a protein involved in aging process and aging-associated diseases. It is predominantly expressed in the kidneys and brain. It plays a pivotal role in mineral metabolism and insulin sensitivity [7], thereby positioning itself as an important biomarker for aging-related health issues. Reduced levels of soluble αKl are associated with several aging-related diseases, such as chronic kidney disease (CKD) [8], osteoporosis [9], atherosclerosis [10], and cognitive decline [11], highlighting its broad clinical relevance. Moreover, αKl is integral to energy balance and glucose homeostasis, influencing processes such as insulin secretion, β-cell health, lipid oxidation in adipose tissue, and hepatic gluconeogenesis [12]. The protein's ability to modulate these processes underscores its potential as a biomarker for metabolic health and its utility in clinical settings. Inflammatory conditions, which downregulate αKl expression, further complicate the pathophysiology of age-related disorders [13], with elevated levels of inflammatory cytokines, like interleukin-6 and TNF-α (tumor necrosis factor-alpha) being inversely related to soluble αKl levels [14, 15]. Notably, central adiposity—a marker of metabolic dysfunction, inflammation, and oxidative stress— has been found to suppressed αKl expression [16, 17].

Given αKl extensive involvement in both aging and metabolic regulation, αKl serves not only as a key indicator of biological aging but also as a potential therapeutic target for managing age-related health conditions, making it a critical focus for public health and clinical research. This broader understanding of αKl 's roles enriches our investigation into body composition metrics beyond the conventional use of BMI. While previous research has predominantly utilized BMI, it may not adequately capture the nuances of fat distribution, inflammation, and metabolic regulation. Our study introduces the BRI as a more precise and accessible metric for assessing central adiposity. Despite the recognized importance of both BRI and αKl in health and disease, whether inflammatory pathways play a vital role in their association remains insufficiently understood. We hypothesize that systemic inflammation may mediate the relationship between BRI and αKl levels. This hypothesis is based on the well-established role of inflammation in linking adiposity to aging-related outcomes [18], as well as the known suppressive effects of pro-inflammatory cytokines on αKl expression [19]. Clarifying the relationship between BRI and αKl may offer deeper insights into the mechanisms underlying obesity-related aging and inform more precise intervention strategies.

In this cross-sectional study, we analyzed data from the National Health and Nutrition Examination Survey (NHANES) to evaluate the relationship between BRI and serum αKl (SαKl) levels. We also assessed the mediating role of inflammatory markers in this association. Our findings aim to elucidate the potential impact of body roundness on aging biomarkers, thereby contributing to a broader understanding of metabolic health and longevity.

## Methods

This cross-sectional study used data from the NHANES, a program conducted by the U.S. Centers for Disease Control and Prevention (CDC) that employs a stratified, multistage probability sampling design to obtain a representative sample of the U.S. civilian, non-institutionalized population. This NHANES-based research was approved by the National Center for Health Statistics ethics review board, with all participants providing written informed consent. The study adhered to the Declaration of Helsinki and complied with STROBE guidelines for observational studies.

### Study participants

Data from five consecutive NHANES cycles (2007–2016) were included, as SαKl measurements were available exclusively for participants aged 40–79 years during this period. From an initial pool of 50,588 participants, individuals not meeting eligibility criteria were sequentially removed. Overall, 31,244 did not fall within the 40–79-year age bracket; 5,580 had missing SαKl data; 587 were missing BRI measurements; 8 were pregnant; 1,530 had a history of cancer; 54 lacked data on inflammatory markers; and 1,627 had missing information for other key variables. Ultimately, 9,958 participants remained in the final sample (Fig. S1).

### BRI definition

BRI was calculated according to the formula developed by Thomas et al. [3], which incorporates waist circumference and height to better estimate body shape and visceral fat distribution:

$$BRI=364.2-365.5\times \sqrt{1-{\left( \frac{waist\;circumference\left(cm\right)}{2\pi } \right)}^{2}\div {\left[0.5\times height\left(m\right)\right]}^{2}}$$

Waist circumference and height were measured by trained examiners at mobile examination centers following standardized NHANES protocols. Standing height was measured using a calibrated stadiometer, and waist circumference was measured at the level of the iliac crest using a flexible tape measure.

### SαKl level

SαKl levels were measured in serum samples collected from participants aged 40–79 years at mobile examination centers, following standardized protocols. Samples were processed, stored at −80 °C, and subsequently shipped to the Northwest Lipid Metabolism and Diabetes Research Laboratories (University of Washington) for analysis between 2019 and 2020, adhering to strict quality control measures [20].

SαKl concentrations were determined using a commercial enzyme-linked immunosorbent assay kit (IBL International, Japan), with all procedures adhering to the manufacturer's instructions. Each sample was analyzed in duplicate to ensure reliability. The average value was used for the final analysis.

### Inflammatory markers

Inflammatory markers were derived from complete blood count data obtained during NHANES physical examinations. Trained phlebotomists drew blood samples using standardized protocols, which were then stored and analyzed in CDC-certified laboratories to ensure accuracy and reliability.

The inflammatory markers included in our study—neutrophil count, lymphocyte count, platelet count, monocyte count, white blood cell (WBC) count, systemic immune inflammation index (SII), neutrophil-to-lymphocyte ratio (NLR), platelet-to-lymphocyte ratio (PLR), and lymphocyte-to-monocyte ratio (LMR) —were selected based on their established roles in assessing systemic inflammation. These markers have been widely used in previous research to evaluate immune responses in chronic diseases, and aging [21–23]. SII was calculated as (platelet count × neutrophil count) / lymphocyte count. It provides a comprehensive measure of systemic inflammation by reflecting the balance between pro-inflammatory immune responses (neutrophils and platelets) and anti-inflammatory (lymphocytes) responses. NLR was computed as the ratio of the absolute neutrophil count to the absolute lymphocyte count, PLR as the ratio of the absolute platelet count to the absolute lymphocyte count, and LMR as the ratio of the absolute lymphocyte count to the absolute monocyte count.

### Covariates

Potential confounders included age; race/ethnicity (Mexican American, other Hispanic, non-Hispanic White, non-Hispanic Black, and Others); marital status (married/living with a partner or living alone), poverty income ratio (PIR) (< 1.30, 1.30 − 2.99, and ≥ 3.00); education level (less than high school, high school/ GED, or above high school); and physical activity level (inactive, moderate, or vigorous). Alcohol intake was categorized as never, former, light-to-moderate, or heavy drinker according to established criteria [24], and smoking status was classified as never, former, or current smoker according to published guidelines [25].

Comorbidities, including diabetes, hypertension, CKD, and CVD, were also taken into consideration. Diabetes and hypertension were identified according to established definitions [26], while CKD and CVD were determined based on self-reported physician diagnoses.

### Statistical analysis

This study utilized 10 years (2007–2016) of NHANES data, with survey weights applied according to NHANES guidelines to ensure national representativeness. Baseline characteristics were summarized using weighted means ± standard errors (SE) for continuous variables and weighted percentages (95% confidence intervals [CI]) for categorical variables. Group differences were assessed using weighted linear regression for continuous variables and the chi-square test for categorical variables. Multivariate linear regression examined the relationship between BRI, inflammatory markers, and SαKl levels.

Three multivariate models were developed. Model 1 was unadjusted. Model 2 adjusted for age, gender, and race/ethnicity. Model 3 included additional adjustments for marital status, PIR, education level, smoking status, alcohol consumption status, physical activity, diabetes, hypertension, CKD, and CVD. BRI was analyzed both as a continuous and categorical variable, with BRI values divided into quartiles (Q1 to Q4) based on population distribution to capture both linear and non-linear associations with SαKl levels. Stratified analyses were also performed to explore the relationship between the BRI and SαKl in subgroups defined by age (< / ≥ 60 years), gender, and comorbidities.

A mediation analysis was conducted to investigate whether inflammatory markers mediated the association between BRI and SαKl level using Model 3. The mediation effect was quantified as the proportion of the indirect effect (path a*b) to the total effect, and its significance was assessed using bootstrapping (5,000 iterations) to obtain the bias-corrected 95% CIs. The mediation was considered significant if the 95% CI did not include zero.

Sensitivity analyses were conducted to ensure the robustness and validity of our findings. To assess potential selection bias, we summarized the characteristics of participants excluded due to missing data and compared them with those included in the study. Furthermore, we standardized the BRI using Z-score transformation and subjected the data to three multivariate models to confirm the stability and reliability. Additionally, we excluded participants with an estimated glomerular filtration rate (eGFR) less than 60 mL/min, calculated using the CKD-EPI formula. This exclusion was based on the premise that severe kidney impairment might confound the relationship between BRI and SαKl levels.

A two-tailed *P* < 0.05 was considered statistically significant. All analyses were performed using R (version 4.3.2) and Free Statistics software (version 1.9). Survey weighting and multivariate regression were operated with the “survey” package, mediation analysis was operated with the “mediation” package.

## Results

### Characteristics

The study included 9,958 participants aged 40–79 years, representing an estimated 82,122,431 individuals from the 2007–2016 cycle (weighted proportions: 51.22% female; mean age: 55.1 years). Table 1 summarizes the weighted population characteristics across BRI quartiles (Q1: 3.43 ± 0.02; Q2: 4.88 ± 0.01; Q3: 6.17 ± 0.01; Q4: 8.89 ± 0.05). Among all participants, the mean BRI was 5.69 ± 0.04, and the mean SαKl level was 848.98 ± 5.41 pg/mL. The racial/ethnic distribution was 6.89% Mexican American, 4.60% other Hispanic, 72.85% Non-Hispanic White, 9.41% Non-Hispanic Black, and 6.25% from other racial individuals.

**Table 1:** Baseline characteristics of the participants in NHANES, 2007 to 2016

Characteristic	Total (*n* = 9958)	Q1 (*n* = 2490)	Q2 (*n* = 2489)	Q3 (*n* = 2489)	Q4 (*n* = 2490)	*P*
**α-klotho (pg/mL)**	848.98 ± 5.41	870.68 ± 8.97	851.03 ± 10.00	831.46 ± 7.52	838.09 ± 7.47	0.003
**Age (years)**	55.07 ± 0.16	52.55 ± 0.27	55.03 ± 0.25	56.45 ± 0.29	56.79 ± 0.26	< 0.001
**BRI**	5.69 ± 0.04	3.43 ± 0.02	4.88 ± 0.01	6.17 ± 0.01	8.89 ± 0.05	< 0.001
**Gender (%)**						< 0.001
Female	51.22 (47.28,55.17)	51.85 (49.18,54.51)	44.51 (41.75,47.28)	48.12 (45.91,50.32)	61.39 (58.74,64.04)	
Male	48.78 (44.98,52.57)	48.15 (45.49,50.82)	55.49 (52.72,58.25)	51.88 (49.68,54.09)	38.61 (35.96,41.26)	
**Race/ethnicity (%)**						< 0.001
Mexican American	6.89 (5.47, 8.32)	3.36 (2.58, 4.13)	6.75 (5.08, 8.42)	9.63 (7.49,11.76)	8.57 (6.11,11.03)	
Other Hispanic	4.60 (3.59, 5.62)	3.53 (2.60,4.46)	4.45 (3.23,5.66)	5.60 (4.11,7.09)	5.06 (3.68,6.44)	
Non-Hispanic White	72.85 (64.80,80.91)	74.72 (71.96,77.48)	73.57 (70.10,77.05)	71.12 (67.10,75.15)	71.52 (67.37,75.67)	
Non-Hispanic Black	9.41 (8.24,10.57)	9.37 (7.91,10.83)	8.25 (6.75, 9.76)	8.98 (7.20,10.75)	11.22 (8.86,13.58)	
Others	6.25 (5.41, 7.08)	9.02 (7.54,10.49)	6.98 (5.40, 8.55)	4.67 (3.55, 5.80)	3.63 (2.39, 4.88)	
**Marital status (%)**						< 0.001
Married/living with partner	70.81 (64.84,76.77)	73.08 (70.79,75.37)	74.02 (71.48,76.56)	71.90 (69.44,74.36)	63.22 (60.13,66.30)	
Living alone	29.19 (27.06,31.32)	26.92 (24.63,29.21)	25.98 (23.44,28.52)	28.10 (25.64,30.56)	36.78 (33.70,39.87)	
**PIR (%)**						< 0.001
Low	17.42 (15.65,19.20)	14.00 (12.04,15.96)	16.09 (13.99,18.19)	18.83 (16.27,21.40)	21.69 (19.24,24.14)	
Middle	26.27 (23.75,28.80)	22.28 (19.73,24.84)	25.03 (22.47,27.59)	26.83 (24.04,29.62)	31.99 (29.53,34.44)	
High	56.31 (50.73,61.88)	63.72 (60.32,67.12)	58.88 (55.18,62.59)	54.34 (50.86,57.81)	46.32 (43.12,49.53)	
**Education level (%)**						< 0.001
Less than high school	16.29 (14.58,17.99)	12.41 (10.17,14.64)	16.05 (14.08,18.02)	18.64 (16.16,21.12)	18.88 (16.49,21.27)	
High school or GED	22.79 (20.31,25.27)	18.87 (16.49,21.25)	22.97 (20.50,25.44)	24.45 (21.76,27.15)	25.65 (23.11,28.19)	
Above high school	60.92 (55.45,66.40)	68.72 (65.26,72.18)	60.98 (57.58,64.37)	56.91 (53.53,60.28)	55.47 (52.55,58.40)	
**Smoking status (%)**						< 0.001
Never	52.49 (48.56,56.42)	56.00 (53.05,58.94)	51.17 (48.19,54.16)	51.53 (48.55,54.52)	50.74 (48.27,53.20)	
Former	28.81 (25.83,31.79)	21.44 (19.35,23.54)	29.69 (26.84,32.54)	32.36 (29.43,35.28)	33.12 (30.26,35.97)	
Current	18.70 (16.97,20.43)	22.56 (19.92,25.20)	19.14 (16.97,21.31)	16.11 (14.67,17.54)	16.15 (13.97,18.32)	
**Alcohol consumption (%)**						< 0.001
Never	10.34 (9.18,11.50)	8.26 (6.81, 9.72)	8.56 (7.32, 9.81)	11.87 (10.12,13.62)	13.36 (11.42,15.30)	
Former	17.54 (16.03,19.05)	12.35 (10.27,14.44)	14.54 (12.88,16.21)	20.66 (18.30,23.01)	24.13 (21.73,26.52)	
Light-to-moderate	55.80 (50.73,60.87)	62.69 (58.84,66.54)	59.81 (57.05,62.58)	50.77 (47.74,53.79)	47.90 (44.64,51.17)	
Heavy	16.32 (14.80,17.85)	16.69 (14.37,19.01)	17.08 (15.18,18.99)	16.71 (14.66,18.76)	14.61 (12.60,16.61)	
**Physical activity (%)**						< 0.001
Inactive	49.00 (44.89,53.11)	35.95 (32.34,39.57)	44.79 (41.54,48.04)	54.30 (51.92,56.69)	64.33 (61.42,67.24)	
Moderate	31.16 (27.90,34.42)	30.68 (27.31,34.05)	33.56 (30.64,36.48)	30.50 (28.11,32.89)	29.64 (26.98,32.30)	
Vigorous	19.84 (17.35,22.33)	33.37 (29.52,37.21)	21.65 (18.86,24.44)	15.19 (13.06,17.33)	6.03 (4.92, 7.13)	
**Diabetes (%)**						< 0.001
No	81.41 (74.84,87.97)	94.17 (93.11,95.22)	87.62 (85.96,89.28)	78.81 (76.37,81.24)	61.37 (58.82,63.92)	
Yes	18.59 (17.20,19.99)	5.83 (4.78, 6.89)	12.38 (10.72,14.04)	21.19 (18.76,23.63)	38.63 (36.08,41.18)	
**Hypertension (%)**						< 0.001
No	53.31 (48.97,57.65)	71.04 (68.52,73.56)	56.66 (53.74,59.59)	48.74 (46.28,51.20)	32.53 (30.13,34.93)	
Yes	46.69 (43.14,50.24)	28.96 (26.44,31.48)	43.34 (40.41,46.26)	51.26 (48.80,53.72)	67.47 (65.07,69.87)	
**CKD (%)**						< 0.001
No	84.91 (78.33,91.50)	89.52 (88.48,90.56)	88.15 (86.09,90.21)	84.85 (83.08,86.61)	75.65 (73.59,77.70)	
Yes	15.09 (13.78,16.39)	10.48 (9.44,11.52)	11.85 (9.79,13.91)	15.15 (13.39,16.92)	24.35 (22.30,26.41)	
**CVD (%)**						< 0.001
No	90.07 (83.25,96.88)	94.52 (93.44,95.60)	92.09 (90.67,93.51)	89.22 (87.43,91.01)	83.18 (81.38,84.98)	
Yes	9.93 (8.98,10.88)	5.48 (4.40, 6.56)	7.91 (6.49, 9.33)	10.78 (8.99,12.57)	16.82 (15.02,18.62)	
**Neutrophil (1000 cell/ul)**	4.25 ± 0.03	3.90 ± 0.05	4.15 ± 0.06	4.30 ± 0.04	4.74 ± 0.05	< 0.001
**Lymphocyte (1000 cell/ul)**	2.06 ± 0.01	1.92 ± 0.02	2.02 ± 0.02	2.13 ± 0.02	2.22 ± 0.02	< 0.001
**Platelet (1000 cell/ul)**	241.78 ± 1.10	237.55 ± 1.96	240.43 ± 1.82	241.86 ± 1.87	248.38 ± 1.46	< 0.001
**Monocyte (1000 cell/ul)**	0.56 ± 0.00	0.52 ± 0.01	0.56 ± 0.01	0.57 ± 0.01	0.60 ± 0.01	< 0.001
**WBC (1000 cell/ul)**	7.12 ± 0.04	6.57 ± 0.06	6.97 ± 0.07	7.26 ± 0.05	7.84 ± 0.06	< 0.001
**SII**	541.19 ± 5.47	522.74 ± 9.27	540.90 ± 10.00	526.74 ± 9.07	578.64 ± 8.47	< 0.001
**NLR**	2.23 ± 0.02	2.18 ± 0.03	2.23 ± 0.04	2.19 ± 0.03	2.32 ± 0.03	0.001
**PLR**	127.87 ± 0.95	134.10 ± 1.52	130.34 ± 1.63	123.46 ± 1.47	121.92 ± 1.23	< 0.001
**LMR**	3.97 ± 0.03	3.97 ± 0.05	3.88 ± 0.04	4.01 ± 0.04	4.03 ± 0.05	0.08

*BRI* Body Roundness Index, *PIR* Poverty income ratio, *CKD* Chronic kidney disease, *CVD* Cardiovascular disease, *WBC* White blood cell, *SII* Systemic immune-inflammatory, *NLR* Neutrophil-to-lymphocyte ratio, *PLR* Platelet-to-lymphocyte ratio, *LMR* Lymphocyte-to-monocyte ratio

Participants with higher BRI were generally older, predominantly female, and more likely to be Mexican American or Non-Hispanic Black, as well as living alone. They also exhibited lower PIR, lower education levels, reduced physical activity, and fewer current smokers. Additionally, they reported lower alcohol consumption and had a higher prevalence of comorbid conditions and elevated inflammation indicators.

### Univariate analysis of SαKl levels

A univariate analysis (Table S1) identified age, BRI, gender, race/ethnicity, smoking status, alcohol consumption, hypertension, CKD, CVD, and inflammatory markers as significantly associated with SαKl levels.

### BRI and SαKl levels

Three models were employed to explore the association between BRI and SαKl levels (Table 2). Across all models, higher BRI was inversely associated with SαKl levels. After fully adjustment, individuals in the highest BRI quartile (Q4) had significantly lower SαKl levels (−38.46 pg/ml) compared to those in the lowest quartile (Q1) (*P* = 0.002).

**Table 2:** Associations between BRI and serum α − klotho levels by multivariate linear regression

	**Model 1**		**Model 2**		**Model 3**	
	**β (95% CI)**	***P***	**β (95% CI)**	***P***	**β (95% CI)**	***P***
**BRI, continuous**	−5.76 (−9.05, −2.47)	< 0.001	−5.94 (−9.32, −2.56)	< 0.001	−7.53 (−11.10, −3.96)	< 0.001
**BRI, categories**						
Q1	Ref		Ref		Ref	
Q2	−19.64 (−44.23, 4.94)	0.122	−12.45 (−37.31, 12.41)	0.329	−12.94 (−37.99, 12.11)	0.316
Q3	−39.21 (−59.27, −19.16)	< 0.001	−32.32 (−52.43, −12.21)	0.002	−36.65 (−56.91, −16.39)	< 0.001
Q4	−32.59 (−53.82, −11.36)	0.004	−31.68 (−53.84, −9.51)	0.007	−38.46 (−61.15, −15.78)	0.002
*P* for trend		< 0.001		0.002		< 0.001
**BRI, per SD**	−13.05 (−20.51, −5.60)	< 0.001	−13.46 (−21.12, −5.80)	< 0.001	−17.06 (−25.15, −8.97)	< 0.001

Model 1: Adjusted for none

Model 2: Adjusted for age, gender, race/ethnicity

Model 3: Adjusted for age, gender, race/ethnicity, marital status, PIR, education level, smoking status, alcohol consumption status, physical activity, diabetes, hypertension, CKD, and CVD

*Abbreviations: CI* Confidence interval, *BRI* body roundness index, *PIR* poverty income ratio, *CKD* chronic kidney disease, *CVD* cardiovascular disease

**Fig. 1:**
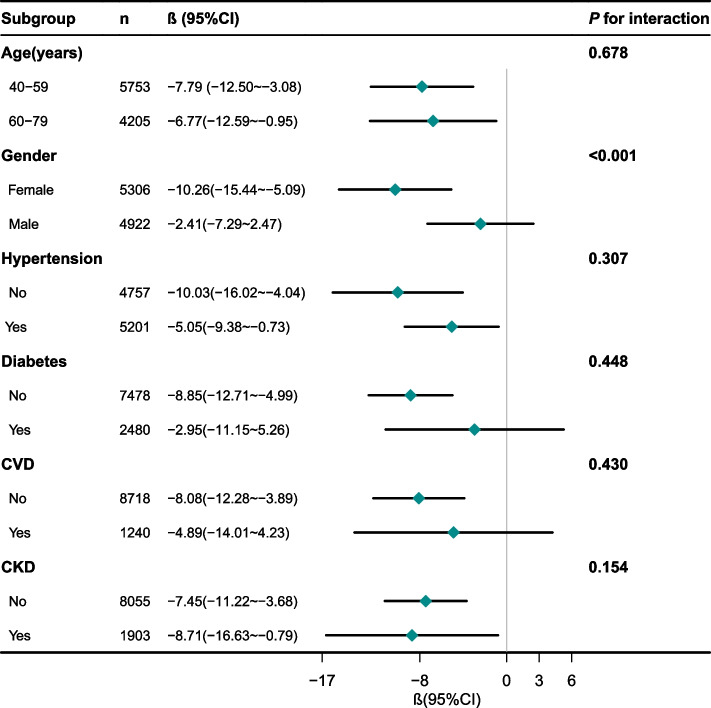
Subgroup analyses of the association between BRI and serum α−Klotho levels Each stratification adjusted for all factors (age, gender, race/ethnicity, marital status, PIR, education level, smoking status, alcohol consumption status, physical activity, diabetes, hypertension, CKD, and CVD) except the stratification factor itself. Abbreviations: PIR, Poverty income ratio; CKD, chronic kidney disease; CVD, cardiovascular disease

Stratified analyses demonstrated that the inverse association between BRI and SαKl remained robust across several subgroups, including individuals aged 40–59 years, 60–79 years, females, and those with or without hypertension or CKD (Fig. 1). In contrast, this association was not statistically significant among males or individuals with diabetes or CVD. A significant interaction by gender (*P* for interaction < 0.001) suggests that the influence of BRI on SαKl may differ from males to females.

### BRI, inflammatory markers and SαKl levels

We utilized three models to examine the relationships between BRI and inflammatory markers (Table 3). In summary, BRI was significantly positively associated with multiple inflammatory markers, including neutrophil count (β = 0.14), lymphocyte count (β = 0.05), platelet count (β = 2.01), monocyte count (β = 0.01), WBC count (β = 0.21), and SII (β = 7.20). In contrast, PLR was negatively associated with BRI (β = −2.25).

**Table 3:** Associations between BRI and inflammation related indicators

	**Model 1**		**Model 2**		**Model 3**	
	**β (95% CI)**	***P***	**β (95% CI)**	***P***	**β (95% CI)**	***P***
Neutrophil	0.15 (0.13, 0.17)	< 0.001	0.17 (0.15, 0.19)	< 0.001	0.14 (0.12, 0.16)	< 0.001
Lymphocyte	0.05 (0.04, 0.06)	< 0.001	0.05 (0.04, 0.06)	< 0.001	0.05 (0.04, 0.06)	< 0.001
Platelet	2.07 (1.32, 2.83)	< 0.001	2.10 (1.36, 2.85)	< 0.001	2.01 (1.18, 2.84)	< 0.001
Monocyte	0.01 (0.01, 0.02)	< 0.001	0.01 (0.01, 0.02)	< 0.001	0.01 (0.01, 0.02)	< 0.001
WBC	0.22 (0.20, 0.25)	< 0.001	0.24 (0.22, 0.27)	< 0.001	0.21 (0.18, 0.23)	< 0.001
SII	10.85 (7.02, 14.67)	< 0.001	11.60 (7.72, 15.48)	< 0.001	7.20 (2.79, 11.61)	0.002
NLR	0.03 (0.02, 0.04)	< 0.001	0.03 (0.02, 0.04)	< 0.001	0.01 (−0.002, 0.03)	0.091
PLR	−1.94 (−2.57, −1.30)	< 0.001	−2.17 (−2.82, −1.53)	< 0.001	−2.25 (−2.91, −1.60)	< 0.001
LMR	0.01 (−0.01, 0.03)	0.443	0.004 (−0.02, 0.02)	0.716	0.01 (−0.01, 0.03)	0.228

Model 1: Adjusted for none

Model 2: Adjusted for age, gender, race/ethnicity

Model 3: Adjusted for age, gender, race/ethnicity, marital status, PIR, education level, smoking status, alcohol consumption status, physical activity, diabetes, hypertension, CKD, and CVD

*Abbreviations: CI* Confidence interval, *WBC* White blood cell, *SII* Systemic immune-inflammatory, *NLR* Neutrophil-to-lymphocyte ratio, *PLR* Platelet-to-lymphocyte ratio, *LMR* Lymphocyte-to-monocyte ratio, *PIR* Poverty income ratio, *CKD* Chronic kidney disease, *CVD* Cardiovascular disease

Similarly, three models evaluated the relationships between inflammatory markers and SαKl level (Table 4). Inflammatory markers, including neutrophil count (β = −10.07), platelet count (β = −0.47), WBC count (β = −7.85), SII (β = −0.05), NLR (β = −8.17), and PLR (β = −0.34) were all negatively associated with SαKl. In contrast, MLR was positively associated with SαKl (β = 3.94).

**Table 4:** Associations between inflammation markers and serum α−klotho levels

	**Model 1**		**Model 2**		**Model 3**	
	**β (95% CI)**	***P***	**β (95% CI)**	***P***	**β (95% CI)**	***P***
Neutrophil	−12.77 (−17.72, −7.82)	< 0.001	−12.07 (−17.08, −7.06)	< 0.001	−10.07 (−15.38, −4.76)	< 0.001
Lymphocyte	−1.94 (−10.31, 6.44)	0.652	−8.82 (−17.33, −0.31)	0.046	−5.26 (−13.98, 3.47)	0.243
Platelet	−0.31 (−0.47, −0.15)	< 0.001	−0.47 (−0.64, −0.31)	< 0.001	−0.47 (−0.64, −0.30)	< 0.001
Monocyte	−75.67 (−118.51, −32.83)	< 0.001	−50.68 (−91.04, −10.31)	0.016	−36.43 (−74.41, 1.55)	0.065
WBC	−9.49 (−13.20, −5.78)	< 0.001	−9.45 (−13.26, −5.66)	< 0.001	−7.85 (−12.04, −3.67)	< 0.001
SII	−0.06 (−0.09, −0.03)	< 0.001	−0.06 (−0.09, −0.03)	< 0.001	−0.05 (−0.08, −0.03)	< 0.001
NLR	−14.90 (−22.18, −7.63)	< 0.001	−10.00 (−17.07, −2.93)	0.007	−8.17 (−14.93, −1.41)	0.021
PLR	−0.29 (−0.45, −0.13)	< 0.001	−0.30 (−0.46, −0.14)	< 0.001	−0.34(−0.50, −0.18)	< 0.001
LMR	10.04 (5.92, 14.17)	< 0.001	3.95 (−0.03, 7.93)	0.052	3.94 (0.15, 7.73)	0.046

Model 1: Adjusted for none

Model 2: Adjusted for age, gender, race/ethnicity

Model 3: Adjusted for age, gender, race/ethnicity, marital status, PIR, education level, smoking status, alcohol consumption status, physical activity, diabetes, hypertension, CKD, and CVD

*CI* Confidence interval, *WBC* White blood cell, *SII* Systemic immune-inflammatory, *NLR* Neutrophil-to-lymphocyte ratio, *PLR* Platelet-to-lymphocyte ratio, *LMR* Lymphocyte-to-monocyte ratio, *PIR* Poverty income ratio, *CKD* Chronic kidney disease, *CVD* Cardiovascular disease

### Mediation effects of inflammatory markers on BRI and SαKl levels associations

**Fig. 2:**
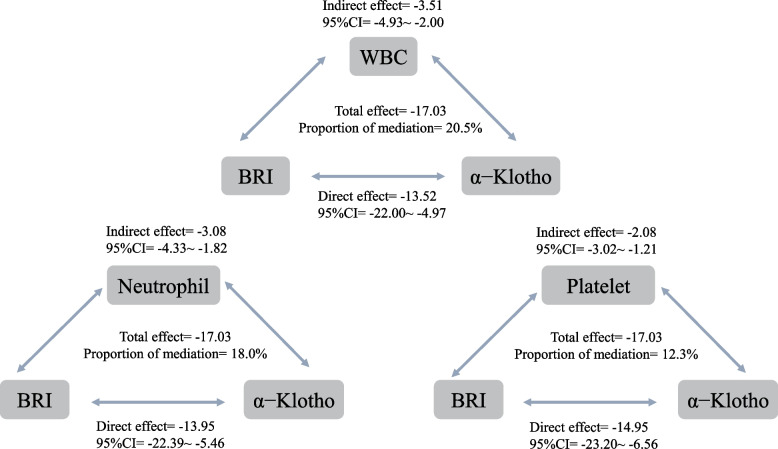
Mediation model of the effect of BRI and serum α−Klotho levels Abbreviations: BRI, Body Roundness Index; CI, Confidence interval; WBC: White blood cell

Given the observed intercorrelations among BRI, inflammatory markers, and SαKl, we performed mediation analyses (Table S2 and Fig. 2). Employing Model 3, we investigated the associations between various inflammatory markers (including neutrophil, lymphocyte, platelet, monocyte, WBC, SII, NLR, PLR, and LMR) and both BRI and SαKl levels. The mediation analyses indicated that WBC count accounted for 20.5% of the association, neutrophil count contributed 18.0%, and platelet count mediated 12.3%.

### Sensitivity analysis

To enhance transparency and address potential selection bias, sensitivity analysises were performed, with the results presented in Table S3. This table provides the characteristics of participants excluded due to missing data, with the most significant exclusions related to PIR and alcohol consumption, which accounted for 6.08% and 5.41% of the potential participant sample, respectively. Other missing data accounted for only 0.42% of the total. Comparisons between excluded and included participants showed no significant differences in key demographic or clinical variables, suggesting minimal bias in our analysis. Additionally, the relationship between BRI and SαKl levels remained consistent when BRI was transformed using the Z-score method, aligning with results from both continuous and categorical analyses (Table 2).

Additionally, after excluding 860 participants with an eGFR less than 60 mL/min, our analysis reaffirmed the robustness of our initial findings across various metrics. The inverse relationship between BRI and SαKl levels remained statistically significant, consistent with the full cohort. Similarly, the associations between BRI, inflammatory markers, and SαKl levels persisted, with inflammatory markers effectively mediating these relationships (Tables S4-S7).

## Discussion

In this cross-sectional study utilizing NHANES data, we identified a significant negative association between BRI and SαKl levels. The negative relationship remained robust after stratification by age, gender, and comorbid conditions (hypertension or CKD), suggesting that these demographic and clinical factors were not fully account for the observed association. Furthermore, we found a significant interaction between gender and SαKl levels, suggesting that central adiposity may influence αKl expression through distinct mechanisms in males and females.

αKl, a protein involved in attenuating mechanisms related to aging, plays a crucial role in mineral metabolism and exerts protective effects against a range of age-related diseases, including CVD [27], osteoporosis [28], and CKD [29]. Its role as a biomarker has significant implications for clinical and public health applications, emphasizes its potential for early detection and intervention in age-related conditions, as well as for identifying individuals at risk of developing obesity and related chronic diseases. Previous research has demonstrated that increased adiposity, particularly visceral fat, is linked to reduced SαKl levels, implying that excessive body fat may inhibit αKl secretion via systemic inflammation and altered adipokine profiles [30, 31]. Given its association with both obesity and metabolic dysfunction, assessing SαKl levels in the general population could help identify individuals at higher risk of future obesity or obesity-related chronic diseases, thereby enabling earlier interventions and more targeted preventive strategies. Age-related decline SαKl levels has also been widely reported, with older individuals generally displaying lower SαKl levels, which is consistent with its role in regulation calcium and phosphate homeostasis and modulates insulin sensitivity, underscoring its pivotal role in metabolic and aging processes [19, 32].

BRI was a newly raised concept that reflect body roundness more accurately. Adiposity is more strongly associated with metabolic derangements and mortality risk than overall adiposity measured by BMI. Our study's findings reinforce these observations, showing that higher BRI values, which incorporate waist circumference and thus more accurately reflect central adiposity, were associated with lower SαKl levels, capturing the complexity of fat distribution's influence on aging and metabolic health. While many earlier studies have utilized BMI to approximate adiposity, we apply BRI to provide a more refined measure of body shape and fat distribution. The distribution of fat is important, as visceral adiposity is more strongly associated with metabolic derangements and mortality risk than overall adiposity measured by BMI. By integrating waist circumference, BRI offers a superior estimation of central adiposity which is more strongly linked to metabolic disturbances and mortality risk [4, 33].

Mechanistically, increased central adiposity fosters systemic inflammation and metabolic stress through several pathways [18, 34]. These include transcriptional regulator interacting with the plant homeodomain zinc finger and/or the bromodomain 2 mediated endoplasmic reticulum stress, non-canonical nuclear factor-kappa B (NF-κB) activation by receptor activator of NF-κB ligand, and disruptions in lipid metabolism. These pathways collectively contribute to the suppression of αKl expression, particularly through lipid metabolism disorders such as inactivation of adenosine 5 ‘-monophosphate-activated protein kinase and altered hormone-sensitive lipase signaling et al. [35–37]. Additionally, visceral adipose tissue induced oxidative stress upregulates of RPS6KB1 (evidenced by increased 8-epi-PGF2α) further exacerbate metabolic dysregulation which may diminish the protective capacity of αKl's [38, 39].

Our findings suggest that inflammatory markers, particularly neutrophils and platelets, play a key role in linking central adiposity to reduced SαKl levels. Elevated neutrophil counts release pro-inflammatory cytokines may disrupting SαKl synthesis, while increased platelets contribute to endothelial dysfunction and oxidative stress, further lowering SαKl [40, 41]. These mechanisms help explain the inverse relationship between BRI and SαKl, further elucidated by the mediating role of inflammation. Furthermore, by identifying specific inflammatory markers that correlate with increased BRI and decreased SαKl levels, our study highlights inflammation as a key mediator. This distinctive aspect of our study provides new insghts into potential therapeutic targets for aging-related conditions, underscoring the clinical relevance of targeting inflammation to modulate the effects of central adiposity on aging biomarker.

Our stratified analyses revealed a stronger inverse association between BRI and SαKl levels among females than in males, highlighting the potential for sex-specific intervention strategies. Recent studies corroborate these findings, with Carreras-Badosa et al. observing that higher SαKl levels are linked to less central obesity in girls [42], and Yin et al. reporting a pronounced inverse relationship between central obesity and SαKl levels in women [43]. These studies emphasize the need to differentiate between central and subcutaneous obesity when examining their effects on SαKl. This divergence may be attributed to sex-specific hormonal differences. Estrogen can promote subcutaneous fat deposition and enhanced αKl expression, potentially buffering females against the adverse effects of central adiposity on SαKl levels [44]. In contrast, androgen worsens visceral fat accumulation in males and may heighten inflammation and metabolic dysfunction, thereby attenuating αKl expression [45]. Moreover, as noted in recent studies, abdominal obesity in women was significantly inversely associated with SαKl levels, particularly among those who developed obesity later in life [42], while no such significant association was found in men. This further suggests that the distribution of fat—particularly visceral fat—plays a key role in influencing SαKl levels, especially in women. This sex-specific divergence underscores the importance of tailored approaches for managing central adiposity and inflammation, particularly in clinical and public health strategies aimed at mitigating age-related health declines.

The inverse association between BRI and SαKl was consistently observed across subgroups with and without hypertension or CKD, indicating that central adiposity influences SαKl levels independently of these conditions. The lack of significance in individuals with diabetes or CVD may stem from the complex interplay of metabolic dysfunction, inflammation, and altered fat distribution characteristic of these conditions. Both diabetes and CVD are associated with chronic low-grade inflammation, which may disrupt SαKl 's regulatory pathways, potentially obscuring its relationship with adiposity [46]. Furthermore, insulin resistance and vascular damage in these populations could further modulate SαKl levels, attenuating the clear inverse association seen in other groups [47].

### Strengths and limitations

This study has several strengths. We leveraged a nationally representative dataset and applied appropriate sampling weights to enhance generalizability, while the comprehensive nature of NHANES facilitated adjustment for multiple confounders. However, several limitations should be considered. The cross-sectional design limits causal inferences between BRI and SαKl levels, and reliance on self-reported data for certain variables may introduce reporting bias. Although BRI provides a more nuanced measure of central adiposity than BMI, it may not encompass all aspects of body composition, and combining additional obesity indices such as body fat percentage could potentially yield a more comprehensive assessment. Additionally, the use of the ELISA technique for measuring αKl, although common, has limitations. Studies suggest that alternative methods such as immunoprecipitation immunoblot might offer more accurate results, especially for samples that have undergone freeze–thaw cycles. Furthermore, the NHANES dataset focuses primarily on serum αKl, with no data on urinary αKl. While serum αKl is widely used in research, urinary αKl may offer a non-invasive alternative, making it easier to assess in clinical practice. Our regression models accounted for key confounders, but residual confounding may still exist. Future research, including longitudinal studies and mechanistic investigations, is needed to confirm these associations, establish causality, and guide interventions aimed at preserving αKl levels and mitigating obesity-related aging phenotypes.

## Conclusions

In conclusion, this study demonstrated a significant inverse association between BRI and SαKl levels, with a pronounced effect observed in females. Mediation analyses further revealed that inflammatory markers substantially influence this relationship, with WBC count, neutrophils, and platelets accounting for 20.5%, 18.0%, and 12.3% of the mediation effect, respectively. These findings suggested that increased adiposity, measured by BRI, was linked to lower SαKl levels, which may potentially contribute to metabolic dysregulation and aging-related diseases through inflammatory pathways.

## Supplementary Information

Supplementary Material 1: Fig S1. Participants selection flowchart. Table S1. Univariate analysis for serum α-klotho level. Table S2. Analysis of the mediation by inflammation-related indicators of the associations of BRI and serum α−klotho levels. Table S3. Baseline characteristics of the participants in NHANES, 2007 to 2016 (including missing data). Table S4. Associations between BRI and serum α−klotho levels by multivariate linear regression (excluded 860 participants with eGFR <60 mL/min). Table S5. Associations between BRI and inflammation markers (excluded 860 participants with eGFR <60 mL/min). Table S6. Associations between inflammation markers and serum α−klotho levels (excluded 860 participants with eGFR <60 mL/min). Table S7. Analysis of the mediation by inflammation-related indicators of the associations of BRI and SαKl levels (excluded 860 participants with eGFR <60 mL/min).

## Data Availability

Data used for this study are available on the NHANES website: https://www.n.cdc.gov/nchs/nhanes/.

## Abbreviations

αKl: α−Klotho
BMI: Body Mass Index
BRI: Body Roundness Index
CDC: Centers for Disease Control and Prevention
CI: Confidence interval
CKD: Chronic kidney disease
CVD: Cardiovascular disease
eGFR: Estimated glomerular filtration rate
NHANES: National Health and Nutrition Examination Survey
PIR: Poverty income ratio
SαKl: Serum α−Klotho
WBC: White blood cell
SII: Systemic immune inflammation index
NLR: Neutrophil-to-lymphocyte ratio
PLR: Platelet-to-lymphocyte ratio
LMR: Lymphocyte-to-monocyte ratio
