# Supplementary material for: Central adiposity and α-klotho: inflammatory mechanisms underlying aging biomarkers related to body roundness index
Source: Lipids Health Dis. 2025 Apr 10;24:136. doi: 10.1186/s12944-025-02541-6 (PMC11984050; doi:10.1186/s12944-025-02541-6)
Supplement: Supplementary file 1 — Supplementary Material 1: Fig S1. Participants selection flowchart. Table S1. Univariate analysis for serum α-klotho level. Table S2. Analysis of the mediation by inflammation-related indicators of the associations of BRI and serum α−klotho levels. Table S3. Baseline characteristics of the participants in NHANES, 2007 to 2016 (including missing data). Table S4. Associations between BRI and serum α−klotho levels by multivariate linear regression (excluded 860 participants with eGFR <60 mL/min). Table S5. Associations between BRI and inflammation markers (excluded 860 participants with eGFR <60 mL/min). Table S6. Associations between inflammation markers and serum α−klotho levels (excluded 860 participants with eGFR <60 mL/min). Table S7. Analysis of the mediation by inflammation-related indicators of the associations of BRI and SαKl levels (excluded 860 participants with eGFR <60 mL/min). [file 12944_2025_2541_MOESM1_ESM.zip › Table S7_ESM.docx]

Table S7 Analysis of the mediation by inflammation-related indicators of the associations of BRI and SαKl levels.

|  | **Mediation effect (95% CI), *P*** | | | |
| --- | --- | --- | --- | --- |
|  | Total effect | Indirect effect | Direct effect | Mediation |
| Neutrophil | -19.02 (-28.33, -9.91) <0.001 | -4.02 (-5.59, -2.40) <0.001 | -15.00 (-24.47, -5.70) <0.001 | 21.0% |
| Lymphocyte | -19.00 (-28.46, -9.99) <0.001 | -0.74 (-1.94, 0.49) 0.240 | -18.26 (-27.69, -9.27) <0.001 | 3.9% |
| Platelet | -19.03 (-28.25, -9.92) <0.001 | -2.31 (-3.36, -1.42) <0.001 | -16.72 (-25.78, -7.88) <0.001 | 12.2% |
| Monocyte | -19.01 (-28.45, -9.96) <0.001 | -1.08 (-2.14, -0.06) 0.042 | -17.93 (-27.26, -8.82) <0.001 | 5.6% |
| WBC | -19.03 (-28.47, -9.90) <0.001 | -4.59 (-6.40, -2.64) <0.001 | -14.44 (-23.76, -5.07) <0.001 | 24.2% |
| SII | -19.03 (-28.30, -10.03) <0.001 | -1.31 (-2.04, -0.69) <0.001 | -17.73 (-27.02, -8.71) <0.001 | 6.9% |
| NLR | -19.01 (-28.51, -9.94) <0.001 | -0.35 (-0.76, -0.05) 0.016 | -18.66 (-28.21, -9.52) <0.001 | 1.8% |
| PLR | -19.00 (-28.24, -10.05) <0.001 | 2.11 (1.30, 3.14) <0.001 | -21.12 (-30.40, -12.08) <0.001 | -11.0% |
| LMR | -18.99 (-28.32, -9.85) <0.001 | 0.08 (-0.06, 0.31) 0.368 | -19.08 (-28.38, -10.06) <0.001 | -0.3% |

Abbreviations: CI, Confidence interval; WBC, White blood cell; SII, Systemic immune-inflammatory; NLR, Neutrophil-to-lymphocyte ratio; PLR, Platelet-to-lymphocyte ratio; LMR, Lymphocyte-to-monocyte ratio.
