# Supplementary material for: Central adiposity and α-klotho: inflammatory mechanisms underlying aging biomarkers related to body roundness index
Source: Lipids Health Dis. 2025 Apr 10;24:136. doi: 10.1186/s12944-025-02541-6 (PMC11984050; doi:10.1186/s12944-025-02541-6)
Supplement: Supplementary file 1 — Supplementary Material 1: Fig S1. Participants selection flowchart. Table S1. Univariate analysis for serum α-klotho level. Table S2. Analysis of the mediation by inflammation-related indicators of the associations of BRI and serum α−klotho levels. Table S3. Baseline characteristics of the participants in NHANES, 2007 to 2016 (including missing data). Table S4. Associations between BRI and serum α−klotho levels by multivariate linear regression (excluded 860 participants with eGFR <60 mL/min). Table S5. Associations between BRI and inflammation markers (excluded 860 participants with eGFR <60 mL/min). Table S6. Associations between inflammation markers and serum α−klotho levels (excluded 860 participants with eGFR <60 mL/min). Table S7. Analysis of the mediation by inflammation-related indicators of the associations of BRI and SαKl levels (excluded 860 participants with eGFR <60 mL/min). [file 12944_2025_2541_MOESM1_ESM.zip › Table S6_ESM.docx]

**Table S6** Associations between inflammation markers and serum α−klotho levels (excluded 860 participants with eGFR < 60 mL/min).

|  | **Model 1** | | **Model 2** | | **Model 3** | |
| --- | --- | --- | --- | --- | --- | --- |
|  | **β (95% CI)** | ***P*** | **β (95% CI)** | ***P*** | **β (95% CI)** | ***P*** |
| Neutrophil | -12.90 (-18.22, -7.58) | <0.001 | -11.88 (-17.28, -6.48) | <0.001 | -10.79 (-16.52, -5.06) | <0.001 |
| Lymphocyte | -0.98 (-11.54, 9.58) | 0.857 | -8.74 (-19.04, 1.55) | 0.100 | -5.39 (-16.89, 6.12) | 0.363 |
| Platelet | -0.31 (-0.48, -0.15) | <0.001 | -0.47 (-0.64, -0.31) | <0.001 | -0.47 (-0.64, -0.30) | <0.001 |
| Monocyte | -71.79 (-108.72, -34.86) | <0.001 | -43.83 (-80.22, -7.45) | 0.021 | -31.65 (-69.04, 5.73) | 0.103 |
| WBC | -9.45 (-13.36, -5.54) | <0.001 | -9.19 (-13.16, -5.22) | <0.001 | -8.26 (-12.83, -3.69) | <0.001 |
| SII | -0.07 (-0.10, -0.05) | <0.001 | -0.07 (-0.10, -0.04) | <0.001 | -0.07 (-0.09, -0.04) | <0.001 |
| NLR | -14.86 (-23.12, -6.60) | <0.001 | -9.95 (-18.07, -1.84) | 0.019 | -9.18 (-16.93, -1.43) | 0.024 |
| PLR | -0.29 (-0.46, -0.13) | <0.001 | -0.31 (-0.48, -0.15) | <0.001 | -0.34(-0.50, -0.18) | <0.001 |
| LMR | 9.41 (5.05, 13.77) | <0.001 | 3.34 (-0.85, 7.55) | 0.122 | 3.50 (-0.55, 7.56) | 0.096 |

Model 1: Adjusted for none.

Model 2: Adjusted for age, gender, race/ethnicity.

Model 3: Adjusted for age, gender, race/ethnicity, marital status, PIR, education level, smoking status, alcohol consumption, physical activity, diabetes, hypertension, CKD, and CVD.

Abbreviations: CI, Confidence interval; WBC, White blood cell; SII, Systemic immune-inflammatory; NLR, Neutrophil-to-lymphocyte ratio; PLR, Platelet-to-lymphocyte ratio; LMR, Lymphocyte-to-monocyte ratio; PIR, Poverty income ratio; CKD, Chronic kidney disease; CVD, Cardiovascular disease.
