# Supplementary material for: Central adiposity and α-klotho: inflammatory mechanisms underlying aging biomarkers related to body roundness index
Source: Lipids Health Dis. 2025 Apr 10;24:136. doi: 10.1186/s12944-025-02541-6 (PMC11984050; doi:10.1186/s12944-025-02541-6)
Supplement: Supplementary file 1 — Supplementary Material 1: Fig S1. Participants selection flowchart. Table S1. Univariate analysis for serum α-klotho level. Table S2. Analysis of the mediation by inflammation-related indicators of the associations of BRI and serum α−klotho levels. Table S3. Baseline characteristics of the participants in NHANES, 2007 to 2016 (including missing data). Table S4. Associations between BRI and serum α−klotho levels by multivariate linear regression (excluded 860 participants with eGFR <60 mL/min). Table S5. Associations between BRI and inflammation markers (excluded 860 participants with eGFR <60 mL/min). Table S6. Associations between inflammation markers and serum α−klotho levels (excluded 860 participants with eGFR <60 mL/min). Table S7. Analysis of the mediation by inflammation-related indicators of the associations of BRI and SαKl levels (excluded 860 participants with eGFR <60 mL/min). [file 12944_2025_2541_MOESM1_ESM.zip › Table S5_ESM.docx]

**Table S5** Associations between BRI and inflammation markers (excluded 860 participants with eGFR < 60 mL/min).

|  | **Model 1** | | **Model 2** | | **Model 3** | |
| --- | --- | --- | --- | --- | --- | --- |
|  | **β (95% CI)** | ***P*** | **β (95% CI)** | ***P*** | **β (95% CI)** | ***P*** |
| Neutrophil | 0.15 (0.13, 0.17) | <0.001 | 0.17 (0.15, 0.19) | <0.001 | 0.14 (0.12, 0.17) | <0.001 |
| Lymphocyte | 0.05 (0.04, 0.06) | <0.001 | 0.05 (0.04, 0.06) | <0.001 | 0.05 (0.04, 0.06) | <0.001 |
| Platelet | 2.19 (1.41, 2.97) | <0.001 | 2.16 (1.38, 2.94) | <0.001 | 1.99 (1.11, 2.87) | <0.001 |
| Monocyte | 0.01 (0.01, 0.02) | <0.001 | 0.01 (0.01, 0.02) | <0.001 | 0.01 (0.01, 0.02) | <0.001 |
| WBC | 0.22 (0.20, 0.25) | <0.001 | 0.24 (0.22, 0.27) | <0.001 | 0.21 (0.19, 0.24) | <0.001 |
| SII | 10.86 (6.54, 15.18) | <0.001 | 11.95 (7.55, 16.35) | <0.001 | 8.36 (3.43, 13.28) | 0.002 |
| NLR | 0.03 (0.01, 0.04) | <0.001 | 0.03 (0.02, 0.05) | <0.001 | 0.02 (0.0004, 0.03) | 0.045 |
| PLR | -1.97 (-2.63, -1.30) | <0.001 | -2.19 (-2.87, -1.51) | <0.001 | -2.23 (-2.92, -1.52) | <0.001 |
| LMR | 0.01 (-0.01, 0.03) | 0.298 | 0.006 (-0.02, 0.03) | 0.617 | 0.01 (-0.01, 0.03) | 0.297 |

Model 1: Adjusted for none.

Model 2: Adjusted for age, gender, race/ethnicity.

Model 3: Adjusted for age, gender, race/ethnicity, marital status, PIR, education level, smoking status, alcohol consumption, physical activity, diabetes, hypertension, CKD, and CVD.

Abbreviations: CI, Confidence interval; WBC, White blood cell; SII, Systemic immune-inflammatory; NLR, Neutrophil-to-lymphocyte ratio; PLR, Platelet-to-lymphocyte ratio; LMR, Lymphocyte-to-monocyte ratio; PIR, Poverty income ratio; CKD, Chronic kidney disease; CVD, Cardiovascular disease.
