# Supplementary material for: Central adiposity and α-klotho: inflammatory mechanisms underlying aging biomarkers related to body roundness index
Source: Lipids Health Dis. 2025 Apr 10;24:136. doi: 10.1186/s12944-025-02541-6 (PMC11984050; doi:10.1186/s12944-025-02541-6)
Supplement: Supplementary file 1 — Supplementary Material 1: Fig S1. Participants selection flowchart. Table S1. Univariate analysis for serum α-klotho level. Table S2. Analysis of the mediation by inflammation-related indicators of the associations of BRI and serum α−klotho levels. Table S3. Baseline characteristics of the participants in NHANES, 2007 to 2016 (including missing data). Table S4. Associations between BRI and serum α−klotho levels by multivariate linear regression (excluded 860 participants with eGFR <60 mL/min). Table S5. Associations between BRI and inflammation markers (excluded 860 participants with eGFR <60 mL/min). Table S6. Associations between inflammation markers and serum α−klotho levels (excluded 860 participants with eGFR <60 mL/min). Table S7. Analysis of the mediation by inflammation-related indicators of the associations of BRI and SαKl levels (excluded 860 participants with eGFR <60 mL/min). [file 12944_2025_2541_MOESM1_ESM.zip › Table S4_ESM.docx]

**Table S4** Associations between BRI and serum α−klotho levels by multivariate linear regression (excluded 860 participants with eGFR < 60 mL/min).

|  | **Model 1** | | **Model 2** | | **Model 3** | |
| --- | --- | --- | --- | --- | --- | --- |
|  | **β (95% CI)** | ***P*** | **β (95% CI)** | ***P*** | **β (95% CI)** | ***P*** |
| **BRI, continuous** | -4.66 (-7.96, -1.37) | <0.001 | -5.34 (-8.81, -1.86) | 0.003 | -8.22 (-11.95, -4.50) | <0.001 |
| **BRI, categories** |  |  |  |  |  |  |
| Q1 | Ref |  | Ref |  | Ref |  |
| Q2 | -26.14 (-52.65, 0.36) | 0.053 | -19.80 (-46.22, 6.63) | 0.146 | -20.72 (-48.40, 6.95) | 0.139 |
| Q3 | -43.01 (-64.95, -21.08) | <0.001 | -38.43 (-60.29, -16.58) | <0.001 | -44.74 (-67.60, -21.89) | <0.001 |
| Q4 | -33.59 (-55.05, -12.13) | 0.003 | -35.83 (-58.05, -13.61) | 0.002 | -50.05 (-74.48, -25.63) | <0.001 |
| *P* for trend |  | <0.001 |  | <0.001 |  | <0.001 |

Model 1: Adjusted for none.

Model 2: Adjusted for age, gender, race/ethnicity.

Model 3: Adjusted for age, gender, race/ethnicity, marital status, PIR, education level, smoking status, alcohol consumption, physical activity, diabetes, hypertension, CKD, and CVD.

Abbreviations: CI, Confidence interval; BRI, body roundness index; PIR, poverty income ratio; CKD, chronic kidney disease; CVD, cardiovascular disease.
