# Supplementary material for: Central adiposity and α-klotho: inflammatory mechanisms underlying aging biomarkers related to body roundness index
Source: Lipids Health Dis. 2025 Apr 10;24:136. doi: 10.1186/s12944-025-02541-6 (PMC11984050; doi:10.1186/s12944-025-02541-6)
Supplement: Supplementary file 1 — Supplementary Material 1: Fig S1. Participants selection flowchart. Table S1. Univariate analysis for serum α-klotho level. Table S2. Analysis of the mediation by inflammation-related indicators of the associations of BRI and serum α−klotho levels. Table S3. Baseline characteristics of the participants in NHANES, 2007 to 2016 (including missing data). Table S4. Associations between BRI and serum α−klotho levels by multivariate linear regression (excluded 860 participants with eGFR <60 mL/min). Table S5. Associations between BRI and inflammation markers (excluded 860 participants with eGFR <60 mL/min). Table S6. Associations between inflammation markers and serum α−klotho levels (excluded 860 participants with eGFR <60 mL/min). Table S7. Analysis of the mediation by inflammation-related indicators of the associations of BRI and SαKl levels (excluded 860 participants with eGFR <60 mL/min). [file 12944_2025_2541_MOESM1_ESM.zip › Table S3_ESM.docx]

**Table S3.** Baseline characteristics of the participants in NHANES, 2007 to 2016

| **Characteristic** | **Total (n=11585)** | **Q1 (n=2896)** | **Q2 (n=2896)** | **Q3 (n=2896)** | **Q4 (n=2897)** | ***P*** |
| --- | --- | --- | --- | --- | --- | --- |
| **α-klotho (pg/mL)** | 851.96±5.23 | 876.58±8.63 | 850.38±9.21 | 835.13±7.47 | 841.06±7.25 | <0.001 |
| **Age (years)** | 55.05±0.15 | 52.55±0.26 | 55.01±0.25 | 56.40±0.28 | 56.76±0.23 | <0.001 |
| **BRI** | 5.67±0.04 | 3.40±0.02 | 4.86±0.01 | 6.14±0.01 | 8.87±0.05 | <0.001 |
| **Gender (%)** |  |  |  |  |  | <0.001 |
| Female | 51.72(48.00,55.44) | 52.35(49.92,54.79) | 44.05(41.49,46.62) | 49.14(46.98,51.29) | 62.44(59.94,64.95) |  |
| Male | 48.28(44.71,51.86) | 47.65(45.21,50.08) | 55.95(53.38,58.51) | 50.86(48.71,53.02) | 37.56(35.05,40.06) |  |
| **Race/ethnicity (%)** |  |  |  |  |  | <0.001 |
| Mexican American | 7.38(5.92, 8.83) | 3.60(2.80, 4.41) | 7.45(5.68, 9.23) | 10.07(7.91,12.24) | 9.11(6.64,11.57) |  |
| Other Hispanic | 5.14(4.06, 6.22) | 3.94(2.93,4.95) | 4.97(3.73,6.21) | 6.33(4.82,7.85) | 5.57(4.15,6.99) |  |
| Non-Hispanic White | 70.96(63.24,78.68) | 73.19(70.43,75.95) | 71.61(67.99,75.23) | 68.90(64.93,72.86) | 69.60(65.35,73.85) |  |
| Non-Hispanic Black | 9.73(8.54,10.92) | 9.47(8.02,10.91) | 8.50(7.00,10.01) | 9.30(7.54,11.07) | 11.90(9.57,14.23) |  |
| Others | 6.80(5.90, 7.70) | 9.80(8.27,11.33) | 7.46(5.90, 9.03) | 5.39(4.23, 6.56) | 3.81(2.67, 4.96) |  |
| **Marital status (%)** |  |  |  |  |  | <0.001 |
| Married/living with partner | 70.77(65.17,76.36) | 72.92(70.83,75.02) | 74.48(72.17,76.79) | 71.59(69.37,73.82) | 63.02(60.22,65.82) |  |
| Living alone | 29.19(27.20,31.17) | 27.05(24.96,29.14) | 25.50(23.19,27.81) | 28.28(26.07,30.49) | 36.98(34.18,39.78) |  |
| Missing | 0.04(-0.01, 0.10) | 0.03(-0.01,0.07) | 0.01(-0.01,0.04) | 0.13(-0.10,0.36) | 0.00(0.00,0.00) |  |
| **PIR (%)** |  |  |  |  |  | <0.001 |
| Low | 16.73(15.10,18.35) | 13.83(12.05,15.60) | 14.88(13.00,16.75) | 18.01(15.68,20.35) | 21.07(18.67,23.47) |  |
| Middle | 24.57(22.25,26.89) | 20.78(18.38,23.18) | 23.52(21.16,25.88) | 24.32(21.74,26.90) | 30.66(28.31,33.00) |  |
| High | 52.63(47.51,57.75) | 59.38(56.08,62.68) | 55.21(51.61,58.81) | 50.68(47.62,53.75) | 43.42(40.21,46.62) |  |
| Missing | 6.08(5.43, 6.72) | 6.02(5.01,7.02) | 6.39(5.29,7.48) | 6.98(5.70,8.27) | 4.86(4.05,5.67) |  |
| **Education level (%)** |  |  |  |  |  | <0.001 |
| Less than high school | 17.38(15.67,19.09) | 13.05(10.98,15.12) | 17.11(15.18,19.03) | 19.83(17.34,22.33) | 20.43(18.16,22.70) |  |
| High school or GED | 22.56(20.18,24.94) | 19.18(16.90,21.45) | 22.18(19.95,24.41) | 24.12(21.74,26.50) | 25.51(23.15,27.87) |  |
| Above high school | 60.03(54.96,65.11) | 67.70(64.35,71.04) | 60.69(57.48,63.89) | 56.03(53.04,59.03) | 54.05(51.26,56.85) |  |
| Missing | 0.03(0.00, 0.06) | 0.07(-0.02,0.16) | 0.02(-0.01,0.06) | 0.01(-0.01,0.02) | 0.01(-0.01,0.02) |  |
| **Smoking status (%)** |  |  |  |  |  | <0.001 |
| Never | 52.87(49.18,56.56) | 55.77(53.09,58.46) | 51.64(48.92,54.36) | 52.45(49.65,55.24) | 51.20(48.97,53.42) |  |
| Former | 28.34(25.64,31.05) | 21.05(19.15,22.94) | 29.47(26.97,31.98) | 31.33(28.45,34.22) | 32.84(30.17,35.51) |  |
| Current | 18.74(17.04,20.45) | 23.16(20.77,25.55) | 18.86(16.71,21.01) | 16.09(14.71,17.46) | 15.96(14.04,17.88) |  |
| Missing | 0.04(-0.01, 0.09) | 0.02(-0.01,0.05) | 0.02(-0.01,0.06) | 0.13(-0.08,0.35) | 0.00(0.00,0.00) |  |
| **Alcohol consumption (%)** |  |  |  |  |  | <0.001 |
| Never | 10.15(9.04,11.25) | 7.94(6.55, 9.34) | 8.60(7.46, 9.74) | 11.48(9.98,12.98) | 13.24(11.42,15.06) |  |
| Former | 16.69(15.26,18.13) | 12.05(10.07,14.03) | 13.78(12.24,15.33) | 18.66(16.42,20.89) | 23.68(21.45,25.92) |  |
| Light-to-moderate | 52.37(47.75,56.99) | 57.79(54.12,61.46) | 56.40(53.66,59.13) | 48.91(46.03,51.80) | 44.67(41.71,47.62) |  |
| Heavy | 15.39(13.98,16.80) | 15.36(13.27,17.46) | 16.10(14.28,17.92) | 15.92(14.07,17.76) | 14.05(12.18,15.92) |  |
| Missing | 5.41(4.64, 6.17) | 6.86(5.49,8.23) | 5.12(3.82,6.41) | 5.03(3.96,6.10) | 4.35(3.33,5.38) |  |
| **Physical activity (%)** |  |  |  |  |  | <0.001 |
| Inactive | 49.00 (44.89,53.11) | 35.95 (32.34,39.57) | 44.79 (41.54,48.04) | 54.30 (51.92,56.69) | 64.33 (61.42,67.24) |  |
| Moderate | 31.16 (27.90,34.42) | 30.68 (27.31,34.05) | 33.56 (30.64,36.48) | 30.50 (28.11,32.89) | 29.64 (26.98,32.30) |  |
| Vigorous | 19.84 (17.35,22.33) | 33.37 (29.52,37.21) | 21.65 (18.86,24.44) | 15.19 (13.06,17.33) | 6.03 (4.92, 7.13) |  |
| **Diabetes (%)** |  |  |  |  |  | <0.001 |
| No | 81.41 (74.84,87.97) | 94.17 (93.11,95.22) | 87.62 (85.96,89.28) | 78.81 (76.37,81.24) | 61.37 (58.82,63.92) |  |
| Yes | 18.59 (17.20,19.99) | 5.83 (4.78, 6.89) | 12.38 (10.72,14.04) | 21.19 (18.76,23.63) | 38.63 (36.08,41.18) |  |
| **Hypertension (%)** |  |  |  |  |  | <0.001 |
| No | 53.62(49.52,57.72) | 71.61(69.32,73.90) | 56.91(54.12,59.69) | 48.71(46.43,50.99) | 32.96(30.86,35.07) |  |
| Yes | 46.38(43.02,49.73) | 28.38(26.09,30.67) | 43.09(40.31,45.88) | 51.29(49.01,53.57) | 67.04(64.93,69.14) |  |
| Missing | 0.00(0.00, 0.01) | 0.01(-0.01,0.04) | 0.00(0.00,0.00) | 0.00(0.00,0.00) | 0.00(0.00,0.00) |  |
| **CKD (%)** |  |  |  |  |  | <0.001 |
| No | 84.86(78.67,91.05) | 89.56(88.52,90.60) | 87.86(86.04,89.69) | 84.79(82.99,86.59) | 75.74(73.86,77.62) |  |
| Yes | 14.84(13.63,16.05) | 9.90(8.90,10.90) | 12.00(10.16,13.84) | 15.07(13.30,16.84) | 23.90(22.01,25.79) |  |
| Missing | 0.30(0.16, 0.44) | 0.54(0.20,0.89) | 0.13(0.01,0.26) | 0.14(0.00,0.28) | 0.36(0.08,0.63) |  |
| **CVD (%)** |  |  |  |  |  | <0.001 |
| No | 90.23(83.85,96.60) | 94.69(93.76,95.62) | 91.82(90.41,93.22) | 89.44(87.83,91.05) | 83.76(82.14,85.37) |  |
| Yes | 9.77(8.83,10.70) | 5.31(4.38, 6.24) | 8.18(6.78, 9.59) | 10.55(8.95,12.16) | 16.22(14.61,17.83) |  |
| Missing | 0.01(0.00, 0.02) | 0.00(0.00,0.00) | 0.00(0.00,0.00) | 0.01(-0.01,0.02) | 0.02(-0.02,0.06) |  |
| Neutrophil (1000 cell/ul) | 4.25±0.03 | 3.90±0.05 | 4.15±0.06 | 4.30±0.04 | 4.74±0.05 | <0.001 |
| Lymphocyte (1000 cell/ul) | 2.06±0.01 | 1.92±0.02 | 2.02±0.02 | 2.13±0.02 | 2.22±0.02 | <0.001 |
| Platelet (1000 cell/ul) | 241.78±1.10 | 237.55±1.96 | 240.43±1.82 | 241.86±1.87 | 248.38±1.46 | < 0.001 |
| Monocyte (1000 cell/ul) | 0.56±0.00 | 0.52±0.01 | 0.56±0.01 | 0.57±0.01 | 0.60±0.01 | <0.001 |
| WBC (1000 cell/ul) | 7.12±0.04 | 6.57±0.06 | 6.97±0.07 | 7.26±0.05 | 7.84±0.06 | <0.001 |
| SII | 541.19±5.47 | 522.74±9.27 | 540.90±10.00 | 526.74±9.07 | 578.64±8.47 | <0.001 |
| NLR | 2.23±0.02 | 2.18±0.03 | 2.23±0.04 | 2.19±0.03 | 2.32±0.03 | 0.001 |
| PLR | 127.87±0.95 | 134.10±1.52 | 130.34±1.63 | 123.46±1.47 | 121.92±1.23 | <0.001 |
| LMR | 3.97±0.03 | 3.97±0.05 | 3.88±0.04 | 4.01±0.04 | 4.03±0.05 | 0.19 |

Abbreviations: BRI, Body Roundness Index; PIR, Poverty income ratio; CKD, Chronic kidney disease; CVD, Cardiovascular disease; WBC, White blood cell; SII, Systemic immune-inflammatory; NLR, Neutrophil-to-lymphocyte ratio; PLR, Platelet-to-lymphocyte ratio; LMR, Lymphocyte-to-monocyte ratio.
