# Supplementary material for: Central adiposity and α-klotho: inflammatory mechanisms underlying aging biomarkers related to body roundness index
Source: Lipids Health Dis. 2025 Apr 10;24:136. doi: 10.1186/s12944-025-02541-6 (PMC11984050; doi:10.1186/s12944-025-02541-6)
Supplement: Supplementary file 1 — Supplementary Material 1: Fig S1. Participants selection flowchart. Table S1. Univariate analysis for serum α-klotho level. Table S2. Analysis of the mediation by inflammation-related indicators of the associations of BRI and serum α−klotho levels. Table S3. Baseline characteristics of the participants in NHANES, 2007 to 2016 (including missing data). Table S4. Associations between BRI and serum α−klotho levels by multivariate linear regression (excluded 860 participants with eGFR <60 mL/min). Table S5. Associations between BRI and inflammation markers (excluded 860 participants with eGFR <60 mL/min). Table S6. Associations between inflammation markers and serum α−klotho levels (excluded 860 participants with eGFR <60 mL/min). Table S7. Analysis of the mediation by inflammation-related indicators of the associations of BRI and SαKl levels (excluded 860 participants with eGFR <60 mL/min). [file 12944_2025_2541_MOESM1_ESM.zip › Table S2_ESM.docx]

Table S2 Analysis of the mediation by inflammation-related indicators of the associations of BRI and serum α−klotho levels.

|  | **Mediation effect (95% CI), *P*** | | | |
| --- | --- | --- | --- | --- |
|  | Total effect | Indirect effect | Direct effect | Mediation |
| Neutrophil | -17.03 (-25.42, -8.61) <0.001 | -3.08 (-4.33, -1.82) <0.001 | -13.95 (-22.39, -5.46) <0.001 | 18.0% |
| Lymphocyte | -17.02 (-25.45, -8.64) <0.001 | -0.43 (-1.39, 0.55) 0.386 | -16.58 (-25.03, -8.17) <0.001 | 2.5% |
| Platelet | -17.03 (-25.16, -8.50) <0.001 | -2.08 (-3.02, -1.21) <0.001 | -14.95 (-23.20, -6.56) <0.001 | 12.3% |
| Monocyte | -17.02 (-25.34, -8.61) <0.001 | -1.02 (-1.94, -0.14) 0.020 | -16.00 (-24.46, -7.58) <0.001 | 5.9% |
| WBC | -17.03 (-25.54, -8.59) <0.001 | -3.51 (-4.93, -2.00) <0.001 | -13.52 (-22.00, -4.97) <0.001 | 20.5% |
| SII | -17.03 (-25.46, -8.61) <0.001 | -0.48 (-0.95, -0.07) 0.026 | -16.55 (-25.02, -8.15) <0.001 | 2.8% |
| NLR | -17.02 (-25.31, -8.61) <0.001 | -0.27 (-0.61, -0.03) 0.022 | -16.75 (-25.11, -8.36) <0.001 | 1.5% |
| PLR | -17.01 (-25.31, -8.61) <0.001 | 2.20 (1.37, 3.20) <0.001 | -19.20 (-27.68, -10.78) <0.001 | -13.0% |
| LMR | -17.01 (-25.50, -8.73) <0.001 | 0.12 (-0.03, 0.38) 0.150 | -17.13 (-25.55, -8.81) <0.001 | -0.6% |

Abbreviations: CI, Confidence interval; WBC, White blood cell; SII, Systemic immune-inflammatory; NLR, Neutrophil-to-lymphocyte ratio; PLR, Platelet-to-lymphocyte ratio; LMR, Lymphocyte-to-monocyte ratio.
