# Supplementary material for: Central adiposity and α-klotho: inflammatory mechanisms underlying aging biomarkers related to body roundness index
Source: Lipids Health Dis. 2025 Apr 10;24:136. doi: 10.1186/s12944-025-02541-6 (PMC11984050; doi:10.1186/s12944-025-02541-6)
Supplement: Supplementary file 1 — Supplementary Material 1: Fig S1. Participants selection flowchart. Table S1. Univariate analysis for serum α-klotho level. Table S2. Analysis of the mediation by inflammation-related indicators of the associations of BRI and serum α−klotho levels. Table S3. Baseline characteristics of the participants in NHANES, 2007 to 2016 (including missing data). Table S4. Associations between BRI and serum α−klotho levels by multivariate linear regression (excluded 860 participants with eGFR <60 mL/min). Table S5. Associations between BRI and inflammation markers (excluded 860 participants with eGFR <60 mL/min). Table S6. Associations between inflammation markers and serum α−klotho levels (excluded 860 participants with eGFR <60 mL/min). Table S7. Analysis of the mediation by inflammation-related indicators of the associations of BRI and SαKl levels (excluded 860 participants with eGFR <60 mL/min). [file 12944_2025_2541_MOESM1_ESM.zip › Table S1_ESM.docx]

**Table S1.** Univariate analysis for serum α-klotho level (pg/ml)

|  | **β (95% CI)** | ***P*** |
| --- | --- | --- |
| Age | -1.90 (-2.59, -1.2) | <0.001 |
| BRI | -5.76 (-9.05, -2.47) | <0.001 |
| **Gender** |  |  |
| Female | Ref | Ref |
| Male | -39.25 (-53.38, -25.13) | <0.001 |
| **Race/ethnicity** |  |  |
| Mexican American | Ref | Ref |
| Other Hispanic | 22.74 (-8.06, 53.53) | 0.152 |
| Non-Hispanic White | -13.57 (-35.02, 7.89) | 0.219 |
| Non-Hispanic Black | 57.90 (26.62, 89.18) | <0.001 |
| Others | -3.85 (-35.62, 27.92) | 0.813 |
| **Marital status** |  |  |
| Married/living with partner | Ref | Ref |
| Living alone | 14.53(-2.65, 31.71) | 0.101 |
| **PIR** |  |  |
| <1.30 | Ref | Ref |
| 1.30-2.99 | 4.57 (-16.46, 25.60) | 0.671 |
| ≥3.00 | 6.02 (-13.52, 25.57) | 0.548 |
| **Education level** |  |  |
| Less than high school | Ref | Ref |
| High school or GED | -18.69 (-41.41, 4.04) | 0.111 |
| Above high school | 13.80 (-8.45, 36.05) | 0.228 |
| **Smoking status** |  |  |
| Never | Ref | Ref |
| Former | -22.90 (-40.36, -5.44) | 0.012 |
| Current | -47.99 (-67.52, -28.45) | <0.001 |
| **A****lcohol consumption** |  |  |
| Never drinker | Ref | Ref |
| Former drinker | -34.16 (-61.94, -6.38) | 0.018 |
| Light-to-moderate drinker | -37.85 (-62.69, -13.02) | 0.004 |
| Heavy drinker | -89.44 (-118.82, -60.07) | <0.001 |
| **Physical activity** |  |  |
| Inactive | Ref | Ref |
| Moderate | -13.76 (-31.61, 4.09) | 0.135 |
| Vigorous | 18.82 (-4.60, 42.23) | 0.119 |
| **Diabetes** |  |  |
| No | Ref | Ref |
| Yes | 3.09 (-13.97, 20.15) | 0.724 |
| **Hypertension** |  |  |
| No | Ref | Ref |
| Yes | -18.29 (-32.44, -4.15) | 0.013 |
| **CKD** |  |  |
| No | Ref | Ref |
| Yes | -41.14 (-62.49, -19.79) | <0.001 |
| **CVD** |  |  |
| No | Ref | Ref |
| Yes | -50.57 (-68.96, -32.18) | <0.001 |
| Neutrophil | -12.77 (-17.72, -7.82) | <0.001 |
| Lymphocyte | -1.94 (-10.31, 6.44) | 0.652 |
| Platelet | -0.31 (-0.47, -0.15) | <0.001 |
| Monocyte | -75.67 (-118.51, -32.83) | <0.001 |
| WBC | -9.49 (-13.20, -5.78) | <0.001 |
| SII | -0.06 (-0.09, -0.03) | <0.001 |
| NLR | -14.90 (-22.18, -7.63) | <0.001 |
| PLR | -0.29 (-0.45, -0.13) | <0.001 |
| LMR | 10.04 (5.92, 14.17) | <0.001 |

Abbreviations: BRI, Body Roundness Index; PIR, Poverty income ratio; CKD, Chronic kidney disease; CVD, Cardiovascular disease; WBC, White blood cell; SII, Systemic immune-inflammatory; NLR, Neutrophil-to-lymphocyte ratio; PLR, Platelet-to-lymphocyte ratio; LMR, Lymphocyte-to-monocyte ratio.
